# Fanconi Anemia Patients from an Indigenous Community in Mexico Carry a New Founder Pathogenic Variant in *FANCG*

**DOI:** 10.3390/ijms23042334

**Published:** 2022-02-20

**Authors:** Pedro Reyes, Benilde García-de Teresa, Ulises Juárez, Fernando Pérez-Villatoro, Moisés O. Fiesco-Roa, Alfredo Rodríguez, Bertha Molina, María Teresa Villarreal-Molina, Jorge Meléndez-Zajgla, Alessandra Carnevale, Leda Torres, Sara Frias

**Affiliations:** 1 Laboratorio de Citogenética, Instituto Nacional de Pediatría, Ciudad de México 04530, Mexico; reyesj87@gmail.com (P.R.); b.garciadeteresa@gmail.com (B.G.-d.T.); ehatlujf@gmail.com (U.J.); frpvillatoro@gmail.com (F.P.-V.); fiescoroa@facmed.unam.mx (M.O.F.-R.); bertha_molina@yahoo.com.mx (B.M.); 2 Departamento de Medicina Genómica y Toxicología Ambiental, Instituto de Investigaciones Biomédicas, Universidad Nacional Autónoma de México, Apartado Postal 70228, Ciudad de México 04510, Mexico; alfredo.rodriguez@iibiomedicas.unam.mx; 3 Doctorado en Ciencias Biomédicas, Universidad Nacional Autónoma de México, Ciudad de México 04510, Mexico; 4 Maestría y Doctorado en Ciencias Médicas, Odontológicas y de la Salud, Universidad Nacional Autónoma de México, Ciudad de México 04510, Mexico; 5 Instituto Nacional de Pediatría, Ciudad de México 04530, Mexico; 6 Laboratorio de Genómica de Enfermedades Cardiovasculares, Instituto Nacional de Medicina Genómica, Ciudad de México 14610, Mexico; mvillareal@inmegen.gob.mx; 7 Laboratorio de Genómica Funcional del Cáncer, Instituto Nacional de Medicina Genómica, Ciudad de México 14610, Mexico; jmelendez@inmegen.gob.mx; 8 Laboratorio de Enfermedades Mendelianas, Instituto Nacional de Medicina Genómica, Ciudad de México 14610, Mexico; acarnevale@inmegen.gob.mx

**Keywords:** Fanconi anemia, chromosome instability, *FANCG*, splicing, founder pathogenic variant, *Mixe* indigenous group

## Abstract

Fanconi anemia (FA) is a rare genetic disorder caused by pathogenic variants (PV) in at least 22 genes, which cooperate in the Fanconi anemia/Breast Cancer (FA/BRCA) pathway to maintain genome stability. PV in *FANCA*, *FANCC*, and *FANCG* account for most cases (~90%). This study evaluated the chromosomal, molecular, and physical phenotypic findings of a novel founder *FANCG* PV, identified in three patients with FA from the *Mixe* community of Oaxaca, Mexico. All patients presented chromosomal instability and a homozygous PV, *FANCG*: c.511-3_511-2delCA, identified by next-generation sequencing analysis. Bioinformatic predictions suggest that this deletion disrupts a splice acceptor site promoting the exon 5 skipping. Analysis of Cytoscan 750 K arrays for haplotyping and global ancestry supported the Mexican origin and founder effect of the variant, reaffirming the high frequency of founder PV in *FANCG*. The degree of bone marrow failure and physical findings (described through the acronyms VACTERL-H and PHENOS) were used to depict the phenotype of the patients. Despite having a similar frequency of chromosomal aberrations and genetic constitution, the phenotype showed a wide spectrum of severity. The identification of a founder PV could help for a systematic and accurate genetic screening of patients with FA suspicion in this population.

## 1. Introduction

Fanconi anemia (FA) is a rare genetic disorder characterized by chromosomal instability, a high predisposition to physical developmental abnormalities, progressive bone marrow failure, solid tumors, and hematological malignancies [1,2]. Genetic heterogeneity is a striking feature of FA. Germline pathogenic variants (PV) in 22 FANC genes (FANCA to FANCW) have been so far associated with the FA phenotype. The protein products of the *FANC* genes participate in the Fanconi anemia/Breast Cancer (FA/BRCA) pathway, a biochemical network that regulates DNA damage repair in response to DNA interstrand crosslinks, maintains genomic stability during DNA replication, and assists other cellular processes [3,4,5].

A large number of genes and hundreds of unique PV have been associated with the FA phenotype, making allelic and locus heterogeneity the rule. Worldwide, the most frequent disease-causing PV among patients with FA occur in *FANCA* (~64%), *FANCC* (~12%), and *FANCG* (~8%) [6,7]. Recurrent PV have been identified in specific ethnic backgrounds due to a founder effect [8]. Notable examples, explained by high rates of carrier individuals, include the *FANCA* c.295C>T in Spanish Gypsies [9], *FANCC* c.456+4A>T in Ashkenazi Jews [10], and *FANCC* c.67delG in the Mennonite Community [11,12].

Regarding *FANCG* (MIM#602956), founder PV have been reported in Portuguese-Brazilian (c.1077-2A>G), Korean-Japanese (c.307+1G>C), French-Acadian (c.1480+1G>C), and Black South African (c.637_643del) populations [13,14]. *FANCG* gene covers around 6 kilobases (kb) that are mapped to chromosome 9p13; it comprises 14 exons and encodes a 2.6 kb messenger RNA (mRNA) transcript that is translated to a 622 amino acid protein with a molecular weight of 68 kilodaltons [15], this protein has seven tetratricopeptide repeat (TPR) motifs, which are required to mediate its protein-protein interactions [15,16]. FANCG protein is an integral component of the FA-core complex, which is a dynamic E3 ubiquitin ligase protein assembly that catalyzes the mono-ubiquitination of the FANCD2-FANCI heterodimer. This mono-ubiquitination is a critical event in the recruitment of the repair machinery to the DNA lesion as part of the FA/BRCA pathway activation [17,18,19].

Here, we report the novel PV in *FANCG*:c.511-3_511-2delCA, detected in three patients from the *Mixe* indigenous community in Mexico. We used a comprehensive molecular and clinical approach to elucidate the consequences of this PV and we provide evidence for a founder effect.

## 2. Results

### 2.1. Chromosomal Breakage Analysis Confirmed a FA Diagnosis

**Figure 1:**
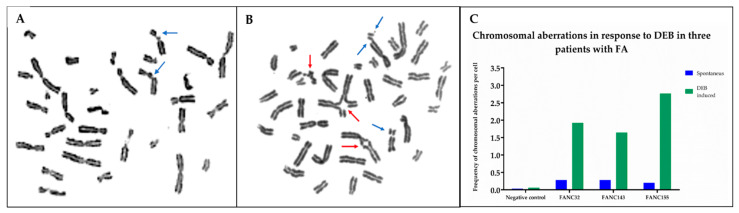
DEB-induced chromosomal aberrations in lymphocytes from healthy and FA individuals. DEB: diepoxybutane. (**A**) Representative metaphase from a healthy individual (negative control); chromosome aberrations are mainly chromatid breaks. (**B**) Representative metaphase from one of the FA patients; increased chromosome aberrations are observed showing chromatid breaks and radial figures, characteristics of patients with FA. Blue arrows show chromatid and isochromatid breaks and red arrows show radial figures. (**C**) Frequency of chromosomal aberrations per cell for each FA patient analyzed in this study.

Chromosomal breakage analysis with diepoxybutane (DEB) was performed in the three patients to support the suspicion of a clinical FA diagnosis. The number of spontaneous and DEB-induced aberrations, as well as the presence of radial chromosomal figures, were considered criteria to establish the diagnosis of FA. In all three cases, the frequency of radial exchange figures and chromatid breaks showed an increase in DEB-induced samples, compared to those recorded in the spontaneous aberration cultures. Hypersensitivity to DEB treatment in patients was similar to that observed in the FA-positive control (VU817 cell line), while induced aberration frequency observed in the patients was higher than the historical average reported in our laboratory for healthy individuals (negative controls). These results confirmed the FA diagnosis in the patients, who were identified as FANC32, FANC143, and FANC155 (Figure 1).

### 2.2. Pathogenic Variant Found in FANCG: c.511-3_511-2delCA Is Predicted to Induce Exon 5 Skipping

Targeted massively parallel DNA sequencing from the three patients with FA revealed a homozygous two-base deletion on *FANCG* (Supplementary Figure S1A and Table 1). This alteration is located 2 base pair (bp) downstream from the nearest acceptor splice site between intron 4 and exon 5. The genotype was subsequently validated via Sanger sequencing (Supplementary Figure S1B). According to gnomAD (https://gnomad.broadinstitute.org/, accessed on 6 December 2021) this variant (rs1491369358) has only been observed in 1/251,480 alleles, corresponding to a worldwide frequency of 0.00000398. Further analysis of the effect of this novel variant, following the recommended American College of Medical Genetics (ACMG) criteria, supports its pathogenicity.

Inspection of the genomic region where the variant was found suggests that this change could disrupt mRNA processing. In silico analysis using The Human Splice Finder tool revealed that *FANCG*:c.511-3_511-2delCA impairs the wild-type acceptor site, most probably affecting splicing. MaxEntScan rated this PV with a score of 1.1, with respect to a reference score of 9.63, predicting a deleterious modification of this splice site. Finally, CRYP—SKYP tool indicated that the deletion may promote exon 5 skipping (probability of cryptic site activation = 0.40). These results consensually predict that this PV in the acceptor splice site leads to loss of *FANCG* exon 5. The consequence of exon 5 skipping would lead to reading frame shift, and generation of a premature stop codon at position p.S171Vfs5*, resulting in a truncated FANCG protein (Supplementary Figure S1C).

### 2.3. Reduced Genetic Variation in Locus g.29754068-38771831 Supporting a Founder Effect for FANCG:c.511-3_511-2delCA in the Mixe Population

The three patients with FA described in this study come from an isolated mountainous region in the state of Oaxaca (southern Mexico). They self-identified as belonging to the *Mixe* indigenous group and shared a surname. Although a kin relationship was unknown, we investigated the possibility of a common haplotype using high-density microarrays (CytoScan 750K array). Several long-contiguous stretches of homozygosity (LCSH) > 3 Mb were shared in autosomal regions by these three patients with FA (Table 2). Some of these LCSH have previously been reported in the HapMap and the 1000 Genomes Project (1KGP) in populations of Asian (CHB), Northern European (CEU) and American (AMR) origin (Supplementary Table S1) [21,22,23,24]. We found two shared zones among the LCSH regions not previously reported. The first one in chromosome 5q spanned 2935 kb (g. 101035497-98100800) and the other one in chromosome 9p spanned 9018 kb (g.29754068-38771831); the latter, importantly, included the *FANCG* locus. Inbreeding coefficient (F), calculated with the total LCSH extension and used to estimate the degree of kinship, suggested a possible parental relationship between the three patients (Table 2).

When we analyzed the g.29754068-38771831 region, we discovered that 439 out of 544 single nucleotide variants (SNV) (80.7%) included in the CytoScan 750K array were common among the three patients. We compared the allelic frequency of these SNVs with the frequency reported in the 1KGP for the global and the Mexican population (MXL). We found that 99.32% of the alleles of our patients are coincident frequencies, compared to the global frequencies and the MXL group, whereas the remaining 0.68% has a different frequency. These results confirm that patients share a common haplotype of 30 SNVs around *FANCG*, supporting the discovery of a founder effect (Supplementary Table S2).

**Figure 2:**
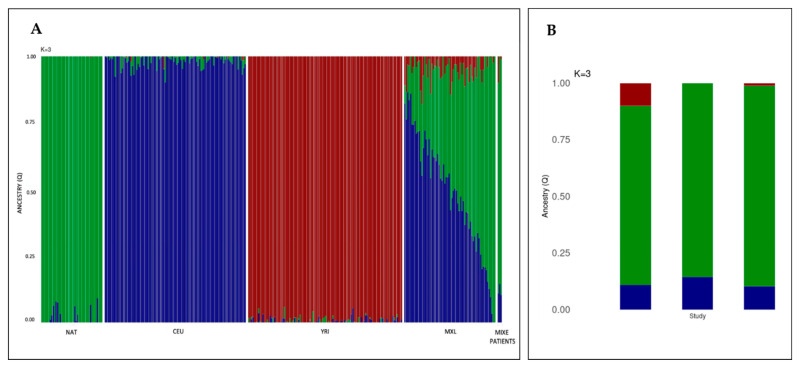
*Mixe* patients with FA present an ancestry pattern that have the hallmarks of the genetic diversity of the Mexican population. (**A**) Barplot showing the inference of global ancestry for the *Mixe* and Mexican individuals using ADMIXTURE, population reference panels include NAT, CEU, and YRI (K = 3 model). Each individual is depicted as a vertical bar. Colors represent the percentage of ancestry assigned to each cluster for each individual. Green, blue, and red colors indicate Native-Americans (NAT), Northern Europeans (CEU), and Yorubas (YRI) populations, respectively. The two panels located at the extreme right of the figure show the three-way ancestry components for the admixed Mexican (MXL) population and the *Mixe* patients (last three bars). (**B**) Detailed bar plot of the three *Mixe* patients with FA, left to right FANC32, FANC143 and FANC155.

Finally, to ascertain that our patients share the Mexican population (MXL) genetic background, as reported in the 1KGP, a global ancestry analysis was performed. Considering that Mexicans present a remarkable genetic diversity due to population admixture, we decided to contrast all SNVs data of these 3 *Mixe* patients against other populations beside the MXL. These populations included the following: Native-Americans (NAT), Utah residents with Northern and Western European ancestry (CEU), and Yoruba in Ibadan, Nigeria (YRI). Through similar approach it has been demonstrated that the MXL population presents an ancestry pattern with Native and European components predominance (NAT: 46.59% and CEU: 48.44%, respectively) [25,26]. Our results indicated that the patients have a proportion of the NAT as high as 79 to 88% (Table 2), with lower CEU and YRI contributions, when compared to the MXL population. Although diversity is observed, the predominance of CEU and NAT ancestry pattern is conserved in both MXL and *Mixe* patients (Figure 2).

### 2.4. The Fraction of Pathogenic Variants Linked to a Founder Effect in FANCG Is the Highest among the Most Common FANC Genes

**Figure 3:**
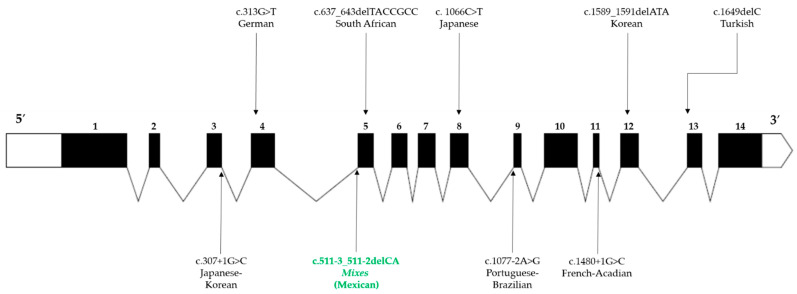
Worldwide founder pathogenic variants reported in *FANCG***.** The structure of *FANCG* is described in 5′-3′ orientation. The untranslated regions are illustrated at the ends of the gene (white areas), the exons are represented by numbered black boxes, and the angled lines correspond to the introns. The location of the variants is indicated by vertical arrows, those located at the bottom of the figure represent splicing variants, while deletions and missense variants are located at the top. The variant described in this study is represented by green letters.

**Figure 4:**
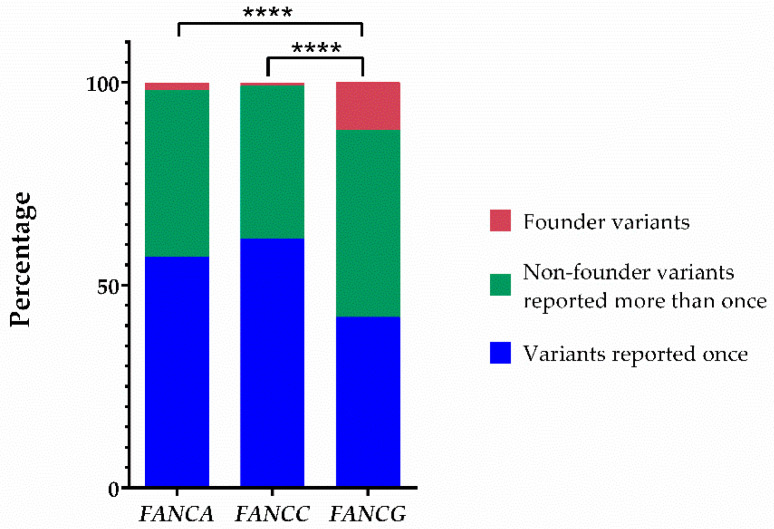
The proportion of variants with founder effect in *FANCG* is the highest among the most frequently reported genotypes. Proportions of founder pathogenic variants, non-founder pathogenic variants reported more than once, and pathogenic variants reported just once in *FANCA*, *FANCC*, and *FANCG*. The significance level of Fisher’s exact test is represented by four asterisks (*p* < 0.0001).

Most founder variants described so far in FA are clustered together in the three most frequently mutated *FANC* genes (i.e., *FANCA*, *FANCC*, and *FANCG*). The PV described here adds a new founder PV (FPV) in *FANCG* (Table 3, Figure 3). The literature analysis indicates that the number of FPV, validated through haplotype analysis, was eleven, three, and eight for *FANCA*, *FANCC*, *FANCG*, respectively; this study adds the ninth FPV in *FANCG* gene. *FANCG* has the lowest number of unique variants among the most frequently affected genes in FA. There were differences between the proportion of founder and non-founder variants among these genes (Figure 4). The Fisher’s exact test to compare the proportion of FPV between *FANCG* and *FANCA* was *p* < 0.0001, whereas between *FANCG* and *FANCC* was *p* < 0.0001 (Figure 4).

### 2.5. Evaluation of the Phenotype of Mixe Patients with FA in the Framework of the Reported FANCG Pathogenic Variant

Medical records of each patient were analyzed to identify physical findings, mainly those described in VACTERL-H (Vertebral, Anal, Cardiac, Tracheo-esophageal fistula, Esophageal atresia, Renal, upper Limb, and Hydrocephalus) association (MIM#192350) and PHENOS (skin Pigmentation, small Head, small Eyes, Nervous system, Otology, Short stature) acronyms [30]. Likewise, hematologic data analysis was used to classify the severity of the bone marrow failure (BMF).

FANC32 patient was the third of four children, born to non-consanguineous parents. His older sister had FA diagnosis and died due to BMF. The patient’s pregnancy was uneventful; he was delivered vaginally at 40 weeks of gestation (WG). At birth his weight was 2500 g (<3rd percentile, HP:0001518) and he had hypotonia (HP:0001252). Developmental milestones were delayed (HP:0012758), beginning to walk at 36 months. He was sent to our clinic at seven years old (y.o.) due to BMF with a history of recurrent upper respiratory tract infections (HP:0002788). Bone marrow aspiration and biopsy performed at age nine y.o. reported 50% cellularity with erythroid series and megakaryocyte predominance and no signs of fibrosis. He was initially treated with steroids and transfusions when required. He later started evaluation for hematopoietic stem-cell transplantation (HSCT); unfortunately, the patient stopped attending follow-up visits. At 14 y.o. anthropometric evaluation showed low weight (−2.31 standard deviation score (SDS), HP:0004325), height (−2.73 SDS, HP:0004322), and head circumference (−2.07 SDS, HP:0000252) for his age and sex. Physical examination revealed thenar hypoplasia of both hands (HP:0001245), generalized hyperpigmentation (HP:0000953), *café au lait* spots (HP:0000957), and melanonychia (HP:0100644). Cardiac and renal structural abnormalities were not found through sonographic evaluation.

FANC143 patient was the first child of non-consanguineous parents. Her pregnancy was complicated by fetal distress (HP:0025116) and oligohydramnios (HP:0001562) which warranted pregnancy termination by C-section at 36 WG. The birth weight was 1590 g (<3rd percentile, HP:0001518), she remained hospitalized her first month of life. Bilateral radial ray alteration (HP:0410049) and left hip dysplasia (HP:0001385) were identified. She had developmental delay (HP:0012758), particularly affecting language (HP:0002463). The patient was referred to our clinic when she was nearly seven y.o. due to BMF. She was initially treated with transfusions when needed and steroids. Physical examination evidenced low weight (−2.15 SDS, HP:0004325), short stature (−2.90 SDS, HP:0004322), and microcephaly (−3.23 SDS, HP:0000252). She had up-slanted palpebral fissures (HP:0000582), bifid uvula (HP:0000193), bilateral microtia (HP:0008551), with folded pinnae of the right ear (HP:0000396), agenesis of the right thumb (HP:0009777), floating hypoplastic left thumb (HP:0009601), generalized skin hyperpigmentation (HP:0000953), and *café au lait* spots (HP:0000957). Tympanometry and brainstem auditory evoked potentials showed right unilateral hearing loss (HP:0000365). Spinal X-rays showed spina bifida in S1 (HP:0004614) and a lumbosacral hemivertebra (HP:0008439). Renal and cardiac malformations were ruled out with imaging methods. Brain magnetic resonance imaging (MRI) revealed cortico-subcortical atrophy (HP:0002120 and HP:0012157), supratentorial ventriculomegaly (HP:0002119), and encephalomalacia (HP:0040197). The patient underwent HSCT at 8.6 y.o. and died a month later due to graft versus host disease complications and septic shock.

FANC155 patient was the younger of two children, born to non-consanguineous parents. Her family health history showed that two great-aunts died of breast cancer in their early thirties. She had a maternal uncle with a history of infertility and vertebral defects. The mother reported an uneventful pregnancy. She was vaginally delivered after 40 weeks, with a birth weight of 2400 g (<3rd percentile, HP:0001518). She had developmental delay (HP:0012758) with language disability (HP:0002463). At six y.o. she was evaluated at a local hospital due to a history of recurrent epistaxis (HP:0004406) and pallor (HP:0000980). After evaluation, a diagnosis of aplastic anemia (HP:0001915) was reached. She received blood transfusions when required and steroid therapy. At 12 y.o. she was referred to our clinic for HSCT. Physical examination evidenced weight (−1.26 SDS) and head circumference (−0.86 SDS) within normal range but low height (−2.18 SDS, HP:0004322) for her age and sex. She had up-slanted palpebral fissures (HP:0000582), ptosis (HP:0000508), simple myopic astigmatism (HP:0500041), hypoplastic helix in both ears (HP:0011039), left hypoplastic thenar region (HP:0001245) with limited ipsilateral thumb flexion, generalized hyperpigmentation (HP:0000953), and *café au lait* spots (HP:0000957). Audiometric findings were unremarkable. Brain MRI, renal ultrasonography, and echocardiography did not show alterations. She underwent HSCT from a non-related donor but died a couple of days after the procedure due to septic shock.

Table 4 summarizes the three patients’ clinical phenotypes. The median age at FA diagnosis was 6.45 y.o. All patients had short stature, skin pigmentation changes, and radial ray anomalies. Respecting VACTERL-H abnormalities, one patient had vertebral and upper limb (radial ray) malformations, and the other two patients had only upper limb alterations; none of them met criteria for VACTERL-H association (≥3/8 features). All three patients had at least three PHENOS anomalies; however, only one had ≥4/6 PHENOS features. Regarding hematologic phenotype, the three patients had moderate to severe BMF.

**Figure 5:**
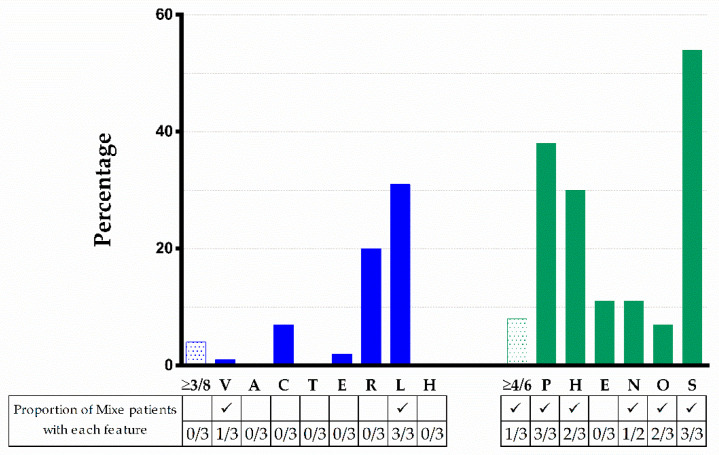
VACTERL-H and PHENOS features in 84 patients from the literature with a *FANCG* genotype and comparison with the three patients with FA here described. Upper limb includes abnormal thumb +/− abnormal radius. Nervous system includes structural brain malformations other than hydrocephalus. Otology comprises ear malformations and/or hearing loss. Solid blue or green bars: VACTERL-H or PHENOS; dotted bars: individual findings. Horizontal axis: abnormalities analyzed; vertical axis: percent of total cases with that abnormality. VACTERL-H association (≥3/8 features) was present in 4% and PHENOS (≥4/6 features) in 8% of patients. The proportion of *Mixe* patients with each feature is shown in the table below the figure. The character ✓ means that this finding was found among *Mixe* patients. Imaging brain evaluation was performed in only two patients.

We compared the physical manifestations observed in the patients reported here to those described in the literature for cases with PV in *FANCG*, we excluded reports in which the phenotypes were not mentioned or individually described. We identified 84 cases with PV in *FANCG*. Remarkably, none of these had any of the 8 founder-effect PV listed in Table 3. The most frequent physical characteristics were short stature (54%), skin pigmentation changes (38%), upper limb (radial ray) abnormalities (31%), microcephaly (29%), and renal malformations (20%). The rest of abnormalities were present in less than 20%. The type and frequency of phenotypic anomalies are depicted in Figure 5. Three out of 84 patients met criteria for VACTERL-H (>3/8 features), seven out of 84 had >4/6 PHENOS features, and only one patient had VACTERL-H plus PHENOS. The median age at diagnosis was 6 y.o. and the median age at report was 9 y.o. The male-to-female ratio was 1:0.64 (*p* = 0.5).

## 3. Discussion

FA is a disorder characterized by high genetic, allelic, and locus heterogeneity. The simultaneous study of several genes using next generation sequencing (NGS)-based targeted panels has advanced the recognition of variants not previously reported within known *FANC* genes. Applying this NGS strategy, we diagnosed three patients from the same geographical region with a classic FA phenotype. We found a non-previously reported PV: *FANCG*:c.511-3_511-2delCA, consisting of a two base-pair deletion affecting the splicing acceptor site of *FANCG* exon 5. This deletion is predicted to lead to an aberrant splicing site and a stop codon, generating a truncated FANCG protein lacking TPR motifs and affecting the function of the FA core complex. Confirmation of a founder effect was obtained by haplotype and ancestry analysis. Overall, our results represent an integrative approach to evaluate the effect of this change in *FANCG* and provide useful information to guide a potential targeted FA screening on the *Mixe* population, a well-defined ethnic group in Mexico.

Besides having the same variant, these patients also share the same geographic and ethnic origin, as well as one of their surnames. These data, in addition to the very low reported frequency of this change (rs1491369358), which has been found in a heterozygous state only in one non-FA individual, led us to explore the possibility of a founder effect for PV *FANCG*:c. 511-3_511-2delCA. The analysis of the degree of inbreeding (F value), the percentage of homozygosity and the regions with LCSH allowed us to corroborate this inference, since all the patients have large stretches of homozygosity in their genomes due to inheritance of identical ancestral genomic segments from both parents. A consequence of this genetic structure is an increased incidence of recessive diseases, like FA. Additionally, the inferred haplotype in the boundaries of the *FANCG* locus supports the hypothesis of a founder effect in this novel PV. Moreover, the high contribution of the native component of around 80% in these three patients contrasts with the average 46% found in the MXL group and supports the idea that these subjects who belong to the *Mixe* group have reduced genetic diversity (Figure 2). While only two of these patients have a native contribution of over 85%, sufficient to name them Mexican natives [31], the third one who does not reach this threshold also has the lower autosomic homozygosity and the more distant familial relationship, reflecting more mixture and illustrating the origin of mestizo-Mexicans [23].

*FANCG*:c.511-3_511-2delCA is the first FPV reported in Mexican patients with FA and is consistent with the demographic history of the *Mixes*; an original population that has resisted several attempts to occupy their lands since the pre-Hispanic era and the Spanish conquest. Even though changes have taken place in the political-administrative organization of the region in which they reside since then, this isolated ethnic group has preserved its language, social structure, and territory [32]. These facts also highlight their sedentary nature, which has led to limited genetic admixture [33]. Considering the appraised size of the community where they come from [34], the calculated prevalence of FA is 7.5/1,000,000, which falls in the upper rank of the estimated worldwide FA prevalence in the general population (1-9/1,000,000) [35]. Of note, FPV constitute more than 10% of the total PV discovered so far in *FANCG*, the highest proportion among the most prevalent *FANC* genes (Figure 4). Although most of the PV in FA are concentrated in *FANCA*, *FANCC*, and *FANCG*, a study suggests that they do not appear to be intrinsically more mutable than the other *FANC* genes [36]; therefore, another explanation should be sought to understand the higher prevalence of PV in these genes. On the other hand, it is unlikely that the rate of FPV in *FANCG* could confer any advantage to carriers, since half of them generate a truncated protein and others a dysfunctional one, so it is likely that they may occur due to chance. Certainly, this observation deserves further investigation.

The patients presented here have a classic FA phenotype, consisting of BMF and physical abnormalities [37]. The acronyms VACTERL-H and PHENOS include the most frequently reported physical abnormalities in patients with FA [30,38]. Among these 14 features, only five are present in over 25% of patients: short stature (43%), radial ray defects (40%), changes in skin pigmentation (37%), microcephaly (27%), and renal malformations (27%) [38]. All three patients described here share height, radial, and skin abnormalities; two also had microcephaly and ultrasonographic evaluations ruled out renal malformations.

Figure 5 shows that 4% of patients reported in the literature with PV in *FANCG* met criteria for VACTERL-H; if we consider all genotypes, this percentage rises to 12% [38]. The percentage of patients reported in the literature with PV in *FANCG* who had ≥4/6 PHENOS features was similar considering all genotypes (8.3 vs. 9%). None of our patients had VACTERL-H and only one had ≥4/6 PHENOS features. None of our patients had evident small eyes, but we did not measure palpebral fissure length, and ocular imaging was not performed. Yet, we did find other ophthalmologic manifestations such as ptosis, myopia, and astigmatism (FANC155). Small eyes are described in 11% of patients with FA regardless of genotype [38], this feature stands for the “E” in the PHENOS acronym. However, the ocular phenotype in FA is wider as recently demonstrated [39] and broadening the features that conform the “E” in PHENOS should be considered.

As expected, although these three patients share the same pathogenic *FANCG* genotype and a similar genetic background, their phenotypic presentation was not homogeneous, as evidenced in other cases [40]. Yet, they illustrate that clinical presentation in FA has a broad spectrum of severity that in this case goes from a florid presentation in FANC143, which includes five PHENOS features and uncommon findings like bifid uvula, to a more discreet presentation in patient FANC155 in whom her unilateral radial abnormality was only recorded after intentional evaluation by a dysmorphologist (Table 4). Concerning the hematologic phenotype, patients with *FANCG* PV have been found to have more severe cytopenia as well as a higher frequency and an earlier diagnosis of acute myeloid leukemia or myelodysplastic syndrome than patients with *FANCA* or *FANCC* genotypes [41]. All three patients have a hematologic phenotype that merited HSCT, yet platelets and neutrophils appeared to be more severely affected than red cells. Oncologic manifestations were not found in these patients, but this observation is certainly limited not only because of the small number of individuals but also due to their young age.

To our knowledge, the only available phenotypic description of a patient cohort with a FPV *FANCG* genotype refers to Black South African individuals, with the homozygous variant c.637_643del [14]. The analysis of 35 patients with this FPV could not definitely establish a particular phenotype for this genotype, yet it was recognized that they had a high frequency of skin pigmentation alterations (97%) and that abnormalities of the upper limbs were frequent (>70%) and subtle (without radial hypoplasia). Also, the frequency of renal abnormalities (37%) was higher than reported elsewhere [42]. A second study that included 24 patients and focused in endocrinologic data, showed that 33%, 46%, and 42% had microcephaly, short stature, and low weight for age, respectively [43]. When comparing the phenotype of the three *Mixe* patients to the Black South African patients, in whom the genotype also leads to a truncated protein (p.Tyr213Lysfs*6), the anthropometric alterations appear to be less severe in the South African patients [43]. We take these observations with caution, both because of the small size of the *Mixe* cohort and the influences of environmental factors and additional *loci* in these multifactorial traits cannot be overlooked. Therefore, a study in this geographical region should be warranted.

In conclusion, in this study we present the ninth FPV in the *FANCG* gene, thereby contributing to reaffirm that this gene has the highest proportion of FPV among the most frequently affected *FANC* genes. Despite having similar genetic and environmental backgrounds, the phenotype of these three *Mixe* patients agrees with the highly variable dysmorphological phenotype of patients with PV in *FANCG* described in the literature. The identification of the founder *FANCG*:c.511-3_511-2delCA PV in these patients constitutes a fundamental step for the systematic and accurate genetic screening of patients with FA in the *Mixe* indigenous group. This could lead to carrier identification, appropriate genetic counseling for families with FA, and optimization of the search for potential bone marrow transplant donors. Finally, the identification of a PV in a population with a high prevalence of the disease represents an opportunity to study the phenotypic effect of the variant in a population with a homogeneous genetic background.

## 4. Materials and Methods

### 4.1. Editorial Policies and Ethical Considerations

This study was approved by the Ethics and Research Committees of the Instituto Nacional de Pediatría (INP) in Mexico City. All patients provided written informed consent to participate in this study (INP 041-2014) and blood samples of each participant were collected by peripheral venipuncture.

### 4.2. Patients

Three patients with cytopenias were independently referred for a medical evaluation to the INP. These patients came from the same geographic region in the southern part of Mexico (Oaxaca State). All patients underwent a comprehensive physical examination by trained medical geneticists. Clinical data including age, sex, parental consanguinity, family history, and anthropometric measurements (weight, height, and head circumference) were recorded. Hematological information, as full blood counts at presentation and subsequent monitoring, bone marrow biopsy results, age of development of bone marrow failure, and transfusions were documented through retrospective review of each patient’s medical file. Available imaging studies were evaluated. For the phenotype description, the Human Phenotype Ontology was used [44].

### 4.3. Chromosomal Breakage Test

A diepoxybutane (DEB) test was performed in all patients. Two-paired lymphocyte cultures per blood sample were cultured in RPMI-1640 medium (Gibco, BRL, Grand Island, NY, USA) and stimulated with phytohemagglutinin (Gibco, BRL, Grand Island, NY, USA). To induce DNA damage, 0.1 μg/mL of DEB (Sigma, St Louis, MO, USA) was added from the start of the incubation to half of the cultures; the untreated cultures were used for the analysis of spontaneous chromosomal breakage. In parallel, cultures of blood samples from healthy individuals as negative controls and the FANCA cell line FA VU817 as a positive control were performed. Harvesting of all cultures was carried out after of 72 h at 37 °C in a 5% CO_2_ incubator. Metaphase spreads and staining were performed according to standard protocols [45]. We evaluated the frequency of chromosome aberrations including breaks, fragments, dicentrics, rings, and radial figures. The frequency of aberrations per cell, as well as the percentage of aberrant cells, were calculated.

### 4.4. Genotyping

#### 4.4.1. Genomic DNA Extraction

The Gentra Puregene Kit (Qiagen, Venlo, Limburg, NL, USA) was used for the extraction of total genomic DNA from peripheral blood samples. The concentrations of the DNA were determined on a Nanodrop spectrophotometer (Nanodrop Technologies, Wilmington, DE, USA) and DNA integrity was verified by agarose gel electrophoresis.

#### 4.4.2. Targeted Next-Generation Sequencing

A customized HaloPlex panel (Agilent Technologies, Santa Clara, CA, USA) was designed to target 16 FA genes (*FANC—A*, *B*, *C*, *D1*, *D2*, *E*, *F*, *G*, *I*, *J*, *L*, *M*, *N*, *O*, *P* and *Q*). The final design included all coding exons and 50 bp of their flanking 5′ and 3′ intronic regions, with a coverage of 99.5%. Agilent’s HaloPlex Enrichment System protocol was followed for library preparation and paired-end sequencing was performed in the MiSeq System platform (Illumina, San Diego, CA, USA). Raw reads were filtered and mapped to the GRCh37/hg19 reference genome with the Burrows-Wheeler Aligner (BWA). The overall mean sequencing depth of the samples was 1000X. Alignment and variant calling were performed using the Genome Analysis Toolkit (GATK), version 4.0.3. A pipeline was designed to maximize the accuracy of variant calls, according to GATK Best Practices recommendations [46,47]. Variants were described following the guidelines proposed by the HGVS nomenclature [48]. The classification of variants, according to the five-tier criteria of the ACMG guidelines [20] as well as the estimated global frequency, were assessed using the online tool VarSome [49].

#### 4.4.3. Sanger Sequencing

*FANCG* variant-specific primers were designed to encompass the splicing acceptor site between intron 4 and exon 5, using the Primer3 software (http://bioinfo.ut.ee/primer3-0.4.0/, accessed on 22 August 2019). The selected primers were as follows: forward 5′- GACCTTGGCGGTAGGCAAA; and reverse 5′- ATTGGGGGAAACTACAGGCA. PCR amplification was carried out from 100 ng of DNA template, according to standard protocols [50]. PCR products were purified with QIAquick kit (QIAGEN, Venlo, Limburg, NL, USA) according to manufacturer instructions. Purified amplicons were bidirectionally sequenced using the Big Dye Terminator sequencing kit and resolved on an Applied Biosystems 3130 Genetic Analyzer (Applied Biosystems, Foster City, CA, USA). The electropherograms were analyzed with Chromas V2.6.6 software (www.technelysium.com.au, accessed on 24 January 2020) and target sequences were compared to the corresponding reference sequences *FANCG* (NG_007312.1) from GenBank (https://www.ncbi.nlm.nih.gov, accessed on 24 January 2020).

### 4.5. In Silico Splice Site Analysis

Human Splicing Finder (HSF) (http://www.umd.be/HSF/, accessed on 5 February 2020, MaxEntScan (http://hollywood.mit.edu/burgelab/maxent/Xmaxentscan_scoreseq_acc.html, accessed on 5 February 2020) and CRYP-SKIP (https://cryp-skip.img.cas.cz/, accessed on 7 February 2020) were the three bioinformatics tools used to predict the effect of the variant on splicing signals.

### 4.6. High-Resolution Microarray Analysis

We performed the analysis of the gDNA with Affymetrix CytoScan 750K Arrays (Affymetrix, Santa Clara, CA, USA) on the three patients. This array provides genome-wide coverage, including 550,000 markers for detecting copy number variation and 200,436 SNP probes. We performed the procedures for DNA digestion, ligation, PCR amplification, fragmentation, labelling, denaturing and hybridization into the array according to the protocols and QC guidelines provided by the supplier. Arrays were then stained and washed in the affymetrix GeneChip Fluidics Station 450 and scanned using an Affymetrix GeneChip Scanner 3000 7G (Affymetrix, Santa Clara, CA, USA); we analyzed the files obtained with the appropriate bioinformatics tools.

#### 4.6.1. Analysis of Long-Contiguous Stretch of Homozygosity

We visualized the LCSH in the software Chromosome Analysis Suite (ChAS) software version 4.1, provided by Affymetrix (Affymetrix, Santa Clara, CA, USA). For the analysis, we used the NetAffx 33 hg19 annotation files (https://www.affymetrix.com/analysis/index.affx, accessed on 20 January 2021). For LCSH > 3Mb, the analysis configuration was set at LOH with marker count = 50 and size = 3000 kb, and for LCSH > 5Mb was set at marker count = 50 and size = 5000 kb.

#### 4.6.2. Estimation of Coefficient of Inbreeding

Individual inbreeding coefficients (F) were estimated using LCSH > 3 Mb data; F was the total length of autosomal LCSH in kb divided by the total autosomal size covered by the Cytoscan 750 array (2,881,033,286 kb for hg19). The F value of 0.25 could reflect a first-degree parental relationship, 0.125 a second-degree, 0.0625 a third-degree and 0.03125 a fourth-degree [51].

#### 4.6.3. Haplotype Inference

We used the LSCH > 5 Mb data to perform a haplotype inference approach, specifically the LCSH region shared by the three patients that include *FANCG*. The number of SNVs identical in the three patients was calculated, and the allele frequency was reviewed to infer the haplotype. The SNVs was compared with those reported in global population and the unrelated Mexican individuals (86 samples, MXL population) found in the 1000 Genomes Project (phase 3) database (1KGP) (http://ftp.1000genomes.ebi.ac.uk/vol1/ftp/phase3/data, accessed on 16 December 2021), using the LDhap tool (https://ldlink.nci.nih.gov/?tab=ldhap, accessed on 16 December 2021).

### 4.7. Ancestry Analysis

For the ancestry study, CEL files derived from the arrays were analyzed with the ChAS software to obtain the SNV genotyping calls, generated from the use of the BRLMM-P-plus algorithm. Subsequently, these results were contrasted with genotype information derived from Affymetrix high-density arrays present in the 1KGP. PLINK 1.9 software (https://zzz.bwh.harvard.edu/plink/download.shtml/, accessed on 25 January 2021) was used to filter and map the SNVs. SNVs with discordant strings or genotypes were corrected or removed. Finally, the global ancestry of the MXL population and the three patients was deducted through a supervised maximum likelihood ADMIXTURE approach, from K = 2 to K = 3 ancestral components. For this analysis, genotypic frequencies of Northern European (CEU), Yoruba (YRI), and Native American (NAT), reported in the 1KGP, were considered as reference parental populations.

### 4.8. Fanconi Anemia Variant Database Analysis

We searched the Fanconi anemia Mutation Database (displayed using Leiden Open Variation Database [LOVD, v.3.0], https://www2.rockefeller.edu/fanconi/genes/, accessed on 30 November 2021) for *FANCA*, *FANCC*, and *FANCG*. We first categorized the variant according to the number of times it has been reported. In those where five or more patients were acknowledged, we reviewed the cited publications in order to establish if a haplotype analysis demonstrated a founder effect. We complemented this with a PubMed/MEDLINE search using the terms: “*FANCA* OR *FANCC* OR *FANCG*” AND “mutation OR variant” AND “founder OR haplotype”. We classified the variants as founder PV (FPV), when demonstrated by haplotype analysis, repeatedly reported non-founder PV (recurrent, but not demonstrated as FPV by haplotype analysis), and those reported just once. We compared the proportion of these variant categories between *FANCG* and the other two genes *FANCA* and *FANCC*. Fisher’s exact test (*p*-values < 0.05) was used to statistically test these differences.

### 4.9. Phenotype Analysis

We searched PubMed/MEDLINE for publications limited to human subjects through 1 December 2020, using the terms “Fanconi” AND “anemia”. We collected and analyzed all cases with a *FANCG* genotype in whom individual patient’s phenotypes were detailed. We described frequencies of VACTERL-H (Vertebral, Anal, Cardiac, Tracheo-esophageal fistula, Esophageal atresia, Renal, upper Limb, and Hydrocephalus) and PHENOS (skin Pigmentation, small Head, small Eyes, Nervous system, Otology, Short stature) features. VACTERL-H association was considered if the patient had ≥3/8 features, and for PHENOS ≥ 4/6 features were needed. We extracted clinical information from publications reporting in *FANCG* FPV (as described in Section 4.8) to compare it to the phenotype of the patients reported here. The statistical analyses were performed using Microsoft Excel Office 365 (Microsoft, Redmond, WA, USA) and R (R Core Team (2013). R: A language and environment for statistical computing. R Foundation for Statistical Computing, Vienna, Austria. http://www.R-project.org/, accessed on 16 December 2021. We used Fisher’s exact test and *p*-values < 0.05 were significant.

## Figures and Tables

**Table 1:** Features of the pathogenic variant found in *FANCG*.

Reference Sequence	DNA Change (Genomic, hg19)	ACMG Criteria Variant Classification	HGVS Nomenclature
NG_007312.1	g.35077398_35077399del	PVS1, PM2, PP3	*FANCG*(NM_004629.2): c.511-3_511-2delCA

Abbreviations: ACMG: American College of Medical Genetics; c.511-3_511-2delCA: two bp deletion in coding DNA; g.35077398_35077399del: two bp deletion in genomic DNA; hg19: Homo sapiens (human) genome assembly GRCh37; HGVS: Human Genome Variation Society; PM2: Pathogenic Moderate 2; PP3: Pathogenic Supporting 3; PVS1: Pathogenic very strong 1. For a complete description of these criteria refer to [20].

**Table 2:** Native component, homozygosity, and coefficient of inbreeding in the three *Mixe* patients with Fanconi anemia.

Patient ID	Total Autosomal LCSH > 3 Mb (kb)	Percentage of Native Component	Autosomal Homozygosity (%)	F	Probable Parental Relationship
FANC32	140214361	79.17%	4.9%	0.0487	Fourth-degree
FANC143	203913825	85.64%	7.1%	0.0708	Third-degree
FANC155	249262167	88.53%	8.7%	0.0865	Third-degree

Abbreviations: F: Inbreeding coefficient; LCSH: Long-contiguous stretches of homozygosity.

**Table 3:** Founder pathogenic variants reported in *FANCG*.

Pathogenic Variant	Location	Effect	Geographic/ Ethnic Background	Reference
c.307+1G > C	Intron 3	Aberrant splicing	Japanese-Korean	[27]
c.313G > T (p.Glu105Ter)	Exon 4	Truncated protein, null variant	German	[28]
c.637_643delTACCGCC (p.Tyr213LysfsTer6)	Exon 5	Truncated protein, null variant	South African	[14]
c.1066C > T (p.Gln356Ter)	Exon 8	Truncated protein, null variant	Japanese	[29]
c.1077-2A > C	Intron 8	Aberrant splicing	Portuguese-Brazilian	[13]
c.1480+1G > C	Intron 11	Aberrant splicing?	French-Acadian	[13]
c.1589_1591delATA (p.Asp530_Thr531delinsAla)	Exon 12	Reduce protein activity, Hypomorphic variant	Korean	[29]
c.1649delC (p.Thr550IlefsTer9)	Exon 13	Truncated protein, null variant	Turkish	[28]
c.511-3_511-2delCA	Intron 4	Aberrant splicing and truncated protein	*Mixe* (Mexican)	Present study

**Table 4:** Summary of the clinical phenotype of the patients described in this study.

Patient ID	Sex	Family History of FA	Age at Diagnosis	Hematologic Phenotype at Initial Evaluation					VACTERL-H Features	PHENOS Features
				Hb (g/dL)	MCV (fL)	ANC (cells/µL)	PLT (cells/µL)	BMF Status		
FANC32	M	Sister	6.45	11.4	100.0	700	26,000	Moderate BMF	L	PHS
FANC143 ^†^	F	No	3.6	8.9	94.1	900	15,000	Moderate BMF	VL	PHNOS
FANC155 ^†^	F	No	9.3	11.1	99.5	220	19,000	Severe BMF	L	POS

Abbreviations: ANC: Absolut Neutrophils Counts; BMF: Bone marrow failure; F: female; Hb: Hemoglobin; FA: Fanconi anemia; M: male; MCV: Mean Corpuscular Volume; PLT: Platelets; PHENOS: skin Pigmentation, small Head, small Eyes, Nervous system, Otology, Short stature; VACTERL-H: Vertebral, Anal, Cardiac, Tracheo-esophageal fistula, Esophageal atresia, Renal, upper Limb, and Hydrocephalus; ^†^ Patient deceased.

## Data Availability

Available upon request, following the approved ethics committee guidelines.

## Supplementary Materials

Supplementary Materials can be found at: https://www.mdpi.com/article/10.3390/ijms23042334/s1.

## Abbreviations

1KGP	1000 Genomes Project
ACMG	American College of Medical Genetics
AMR	Ad Mixed American (1KGP super population)
ANC	Absolut Neutrophils Counts
bp	base pair
BMF	Bone marrow failure
CNS	Central nervous system
CEU	Utah residents (CEPH) with Northern and Western European ancestry (1KGP population)
ChAS	Chromosome Analysis Suite
CHB	Han Chinese in Beijing, China (1KGP population)
DEB	Diepoxibutane
FA	Fanconi anemia
FA/BRCA	Fanconi anemia/Breast Cancer pathway
FPV	Founder pathogenic variant
Hb	Hemoglobin
HGVS	Human Genome Variation Society
HSCT	Hematopoietic stem-cell transplantation
HSF	Human Splicing Finder
Hg19	Homo sapiens (human) genome assembly GRCh37
INP	Instituto Nacional de Pediatría
kb	kilobases
LCSH	Long-contiguous stretches of homozygosity
MCV	Mean Corpuscular Volume
MRI	Magnetic resonance imaging
mRNA	messenger RNA
MXL	Mexican Ancestry in Los Angeles CA, USA (1 KGP population)
MT	Mutant
NAT	Native Americans (1KGP population)
NGS	Next generation sequencing
NMD	Non-sense mediated decay
OMIM	Online Mendelian Inheritance in Man
PCR	Polymerase chain reaction
PHENOS	Skin Pigmentation, small Head, small Eyes, Nervous system, Otology, Short stature
PLT	Platelets
PVS1	Pathogenic Very Strong 1
PM2	Pathogenic Moderate 2
PP3	Pathogenic Supporting 3
PDB	Protein Data Bank
PV	Pathogenic variants
SNV	Single nucleotide variant
SDS	Standard deviation score
TPR	Tetratricopeptide repeat
URTI	Upper respiratory tract infection
VACTERL-H	Vertebral, Anal, Cardiac, Tracheo-esophageal fistula, Esophageal atresia, Renal, upper Limb, and Hydrocephalus
WG	Weeks of gestation
WT	Wild type
y.o.	Year old
YRI	Yoruba in Ibadan, Nigeria (1KGP population)
