# Supplementary material for: Fanconi Anemia Patients from an Indigenous Community in Mexico Carry a New Founder Pathogenic Variant in FANCG
Source: Int J Mol Sci. 2022 Feb 20;23(4):2334. doi: 10.3390/ijms23042334 (PMC8877758; doi:10.3390/ijms23042334)
Supplement: Supplementary file 1 [file ijms-23-02334-s001.zip › Supplementary Figure S1.pdf]

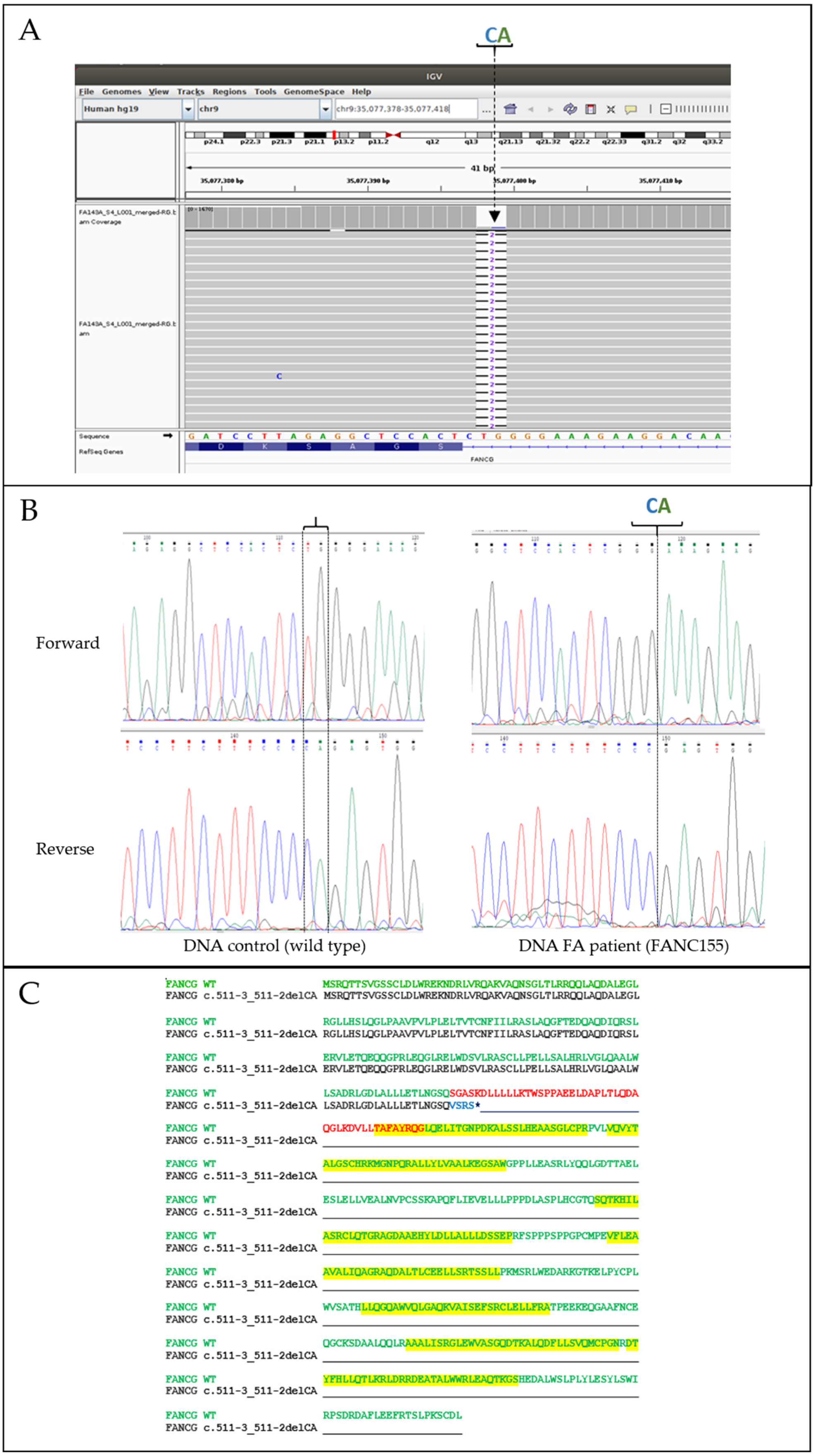

Figure S1. Detection of a novel homozygous PV in *FANCG*. (**A**) The homozygous deletion *FANCG*: c.511-3_511-2delCA was detected by Haloplex-NGS. Visual exploration of this change in FANC143.bam file with the

Integrative Genomics Viewer software (http://software.broadinstitute.org/software/igv/) is displayed. (**B**) PV *FANCG*: c.511-3_511-2delCA was corroborated by bidirectional Sanger sequencing. Representative electropherograms of a healthy control and FANC155 are shown. (**C**) Amino acid sequence resulting from the wild type (WT) protein (Capital letters in green) and mutant protein (*FANCG* c.511-3_511-2delCA) (Capital letters in black). Note that the codified amino acids by exon 5 (Capital letters in red) are missing in the FANCG mutant protein and FVP frmashift generate a stop codon (Capital letters in blue). Yellow highlight indicates the TPR motifs of the WT protein.
