# Supplementary material for: Fanconi Anemia Patients from an Indigenous Community in Mexico Carry a New Founder Pathogenic Variant in FANCG
Source: Int J Mol Sci. 2022 Feb 20;23(4):2334. doi: 10.3390/ijms23042334 (PMC8877758; doi:10.3390/ijms23042334)
Supplement: Supplementary file 1 [file ijms-23-02334-s001.zip › Supplementary Table S1.pdf]

Supplementary Table 1. Long-contiguous stretch of homozygosity (LCSH), previously reported as commons in different population. The three FA patients showed LCSH >3Mb in various autosomes, depicted as present.

FANC32	FANC143	FA155	*Locus*	Coordinates	Size(Mb)	Population	References
Present			1p33p32.3	chr1:49,205,205 - 53,121,054	3.9	California, USA CEU, Hispanic. Florianopolis, Brazil CEU	Kearney/Sanchez, personal communication 2017 in Chaves *et al.,* 2019
Present			1q21.2q21.3	chr1:147,215,796 - 150,287,884	3.1	California, USA CEU, Hispanic. Florianopolis, Brazil CEU	Kearney/Sanchez, personal communication 2017 in Chaves *et al.,* 2019
Present		Present	3p21.33p21.1	chr3:48,787,219 - 50,483,437	1.7	California, USA CEU, Hispanic	Wang *et al*., 2015
		Present	6p22.3p21.31	chr6:26,184,041 - 30,071,279	3.9	Estonia	Pajusalu *et al*., 2015
	Present	Present	7q11.22q11.23	chr7:76,128,151-71,997,278	4.1	Florianopolis, Brazil CEU	Chaves *et al.* 2019
	Present		11p11.2q12.1	chr11:46,342,376 - 51,274,692	4.9	California, USA CEU, hispanic	Wang *et al*., 2015
Present			20q11.21q11.23	chr20:31,940,638 - 36,081,725	4.1	Taipei, Taiwan Han Chinese, Taiwanese, CEU California, USA CEU, Hispanic. Florianopolis, Brazil CEU	Ling-Hui *et al*., 2006/ Kearney/Sanchez, personal communication 2017 in Chaves *et al.,* 2019

Chaves, TF; Oliveira, LF; Ocampos, M; Barbato, IT; de Luca, GR; Filho, JHB; Pinto, LLC; Bernardi, P y Maris, AF. Long contiguous stretches of homozigosity detected by chromosomal microarrays (CMA) in patients with neurodevelopmental disorders in the South of Brazil. BMC Med Genom (2019) 12(50):1-13.

Ling-Hui, L; Sheng-Feng, H; Chien-Hsiun, C; Chun-Yu, W; Wan-Ching, W; Li-Ying, L; Shuen-Iu, H; Wen-Hung, C; Wen-Han, P; et al. Long Contiguous Stretches of Homozygosity in the Human Genome. Hum Mutat (2006) 27(11):1115-1121.

Pajusalu, S; Zilina, O; Yakoreva, M; Tammur, P; Kuuse, K; Mölter-Väär, T; Nôukas, M; Reimand, T y Ôunap, K. The Diagnostic Utility of Single Long Contiguous Stretches of Homozigosity in Patients without Parental Consanguinity. Mol Syndromol (2015) 6:135-140.

Wang, JC; Ross, L; Mahon, LW; Owen, R; Hemmat, M; Wang, BT; Naggar, ME; Kopita, KA; Randolph, LM; Chase, JM; Aguilera, MJM; et al. Regions of homozigosity identified by oligonucleotide SNP arrays: evaluating the incidence and clinical utility. Eur J Hum Genet (2015) 23:663-671.
